# Impacts of interpersonal distancing on-board trains during the COVID-19 emergency

**DOI:** 10.1186/s12544-021-00474-6

**Published:** 2021-02-09

**Authors:** Pierluigi Coppola, Francesco De Fabiis

**Affiliations:** Department of Mechanical Engineering, Polytechnic of Milan, Via G. La Masa 1, 20156 Milano, Italy

**Keywords:** COVID-19, Interpersonal distancing, High-speed rail, Suburban rail services, Demand forecasting, Train capacity

## Abstract

**Introduction:**

The COVID-19 emergency and the cities lockdown have had a strong impact on transport and mobility. In particular, travel demand has registered an unprecedented overall contraction, dramatically dropping down with peaks of - 90%-95% passengers for public transport (PT). During the re-opening phase, demand is gradually resuming the levels before the crisis, although some structural changes are observed in travel behaviour, and containment measures to reduce the risk of contagion are still being applied, affecting transport supply.

**Objective:**

This paper aims at assessing to what extent keeping a one-meter interpersonal distancing on-board trains is sustainable for public transport companies.

**Method:**

The analysis is based on travel demand forecasting models applied to two case-studies in Italy: a suburban railway line and a High-speed Rail (HSR) line, differentiated by demand characteristics (e.g. urban vs. ex-urban) and train access system (free access vs. reservation required).

**Results:**

In the suburban case, the results show the need of new urban policies, not only limited to the transport domain, in order to manage the demand peaks at the stations and on-board vehicles. In the ex-urban case, the outputs suggest the need for public subsidies in order for the railways undertakings to cope with revenue losses and, at the same time, to maintain service quality levels.

## Introduction

Between the end of 2019 and the first months of 2020, the SARS-CoV-2, better known as COVID-19, has rapidly spread from China to Japan, South Korea, Europe, and to the United States. Due to the high number of infections, deaths and hospitalizations caused by the effects of the virus (which primarily causes respiratory infections and cardiovascular disorders), on March 11th, 2020, the World Health Organization (WHO) recognized COVID-19 as a global pandemic [19]. To give an idea of the extent of the spread of the virus, after 1 year, over 88 million cases and 1.9 million deaths have been globally reported [22].

In recent months, scientists from all over the world have studied the virus and worked hard to develop a vaccine. At the same time, numerous countries have adopted a series of non-pharmaceutical public health measures to slow down the spread of the virus. These can be grouped into three different categories [23]:

- Isolation measures, which consist in avoiding any contacts between infected and non-infected people;
- Quarantine measures, which are meant for those who are presumed to have been exposed to the virus;
- Community containment measures, that are provisions applied to an entire community, city or region, designed to reduce personal interactions and movements.

In particular, the latter can range from the obligation to use face masks, to social distancing (for example, banning public meetings and non-essential travel, closure of schools, obligation to work from home), from interpersonal distancing (for example, in university classrooms or on public transport vehicles), up to cities lockdown.

All these measures are having a strong impact on the mobility sector: undergoing structural changes in travel demand are likely to persist in the future [10]. In fact, it is well-know that a decrease in accessibility corresponds to reduced trip generation rates [8]. Moreover, people who are currently working from home due to health emergency restrictions, could also in the future keep managing their business from home, and could be more incline towards e-commerce, reducing the number of their shopping trips [18]. Moreover, in terms of modal choice, users could avoid using public transport (PT) due to the fear of contagion, as it happened in China where, after the first lockdown, travels shifted from PT to private vehicles, with an increase of car usage from 34% to 66% [11].

This paper aims at giving a contribution to the undergoing debate about the impact of the COVID-19 containment measures on mobility, focusing on the interpersonal distancing onboard trains. The research question is: *under which conditions the reduction of line capacity due to the interpersonal distancing is feasible and financially sustainable for railways undertakings*? *When does this measure create discomfort to travelers?*

Two case studies are presented: the first one is related to a suburban railway line with demand peaks in the rush hours; the second one is related to the High-speed Rail (HSR) line between Milan-Rome and Naples, where a reduction of train capacity could lead to revenue losses.

The paper is organized as follows. In Section 2 a focus on interpersonal distancing on-board public transport is presented, showing different views about its effectiveness and sustainability along with the potential problems that may arise for Public Transport. The methodological approach to assess the impacts of interpersonal distancing on the two railway lines considered is described in Section 3. Section 4 presents the simulation results for the two considered case studies. These are discussed in Section 5. Conclusions and future research directions are finally drawn in Section 6.

## Background

The motivation for a study on the impacts of interpersonal distancing on-board suburban and high-speed trains came from a survey among industries, consultancies, and PT companies, about the factors that could most influence transport and mobility due to the health emergency [7]. The survey aimed at testing the effectiveness and economic sustainability of some measures proposed for dealing with the COVID-19 emergency, such as:

- Differentiated fares by time-of-day;
- Seat reservation or reservation of travel-time slots to access stations;
- Use of protection mask and scan of passengers’ body temperature;
- On-board interpersonal distancing and vehicles sanitization;
- Demand-responsive or dedicated home-to-work shuttle services;
- Increasing PT service frequency to improve PT capacity.

To measure how the respondents perceived the effectiveness and sustainability of the above measures, a Likert scale from 1 to 5 was adopted, where 1 corresponds to “very low” effectiveness/sustainability, 2 “low”, 3 “neutral”, 4 “high” and 5 “very high”. Provided that perceptions might vary with respondent’s background and might change due to a learning process during the survey [14], a clear definition of effectiveness and sustainability has been adopted: “Effectiveness” here refers to the capacity of a specific measure to reduce the spread of the contagion, whereas “Sustainability” refers to the ability of the PT operators to implement the proposed measure and to sustain the additional financial and organizational burden.

**Fig. 1:**
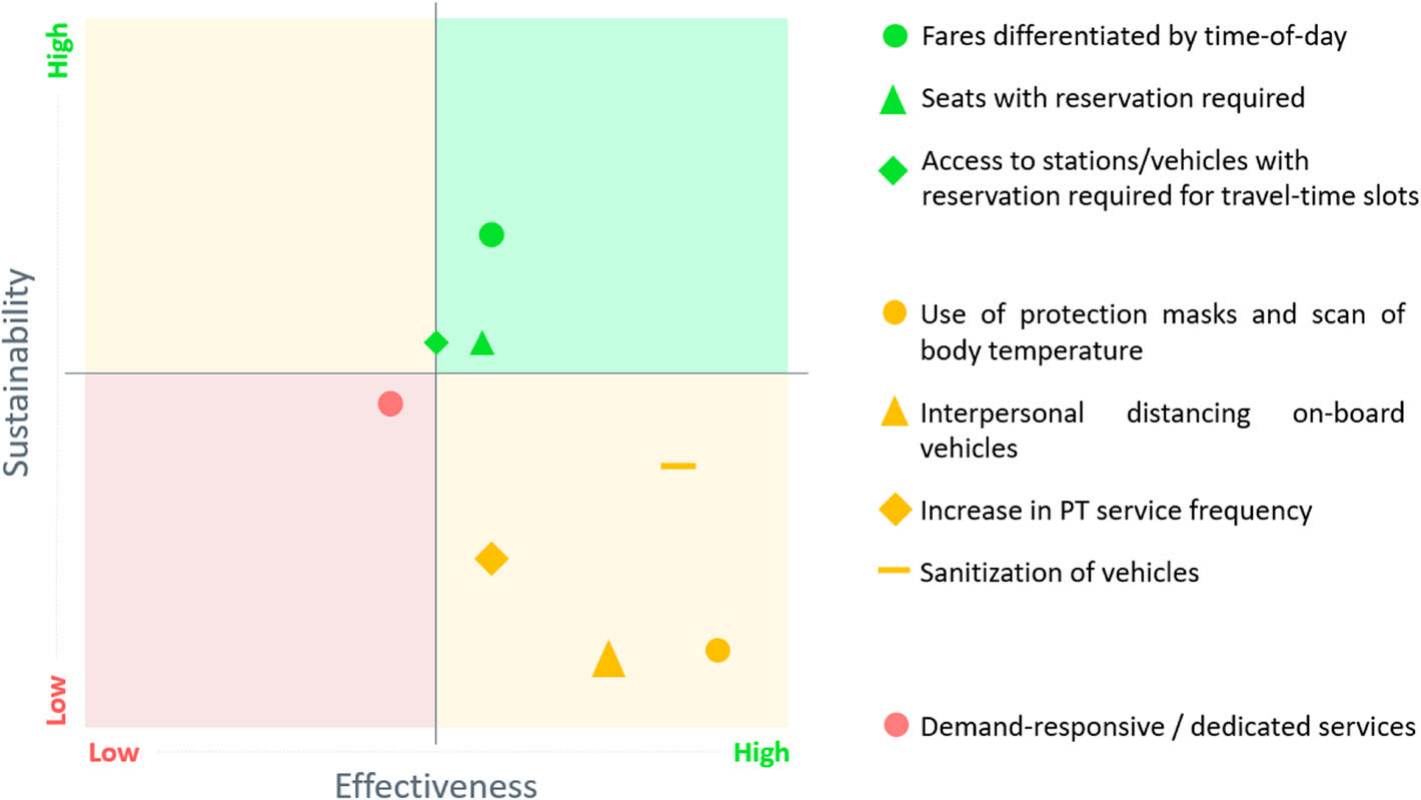
Domains of effectiveness and sustainability of the proposed measures to cope with the COVID-19 health emergency (Source: [7])

**Fig. 2:**
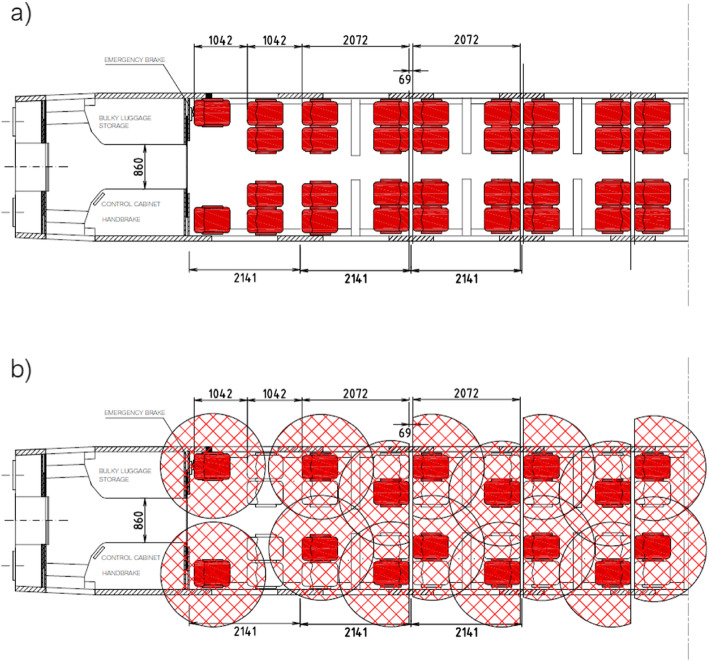
Example of available seats on a High-speed train ETR 500 Standard coach with full occupancy (**a**) and with one-meter interpersonal distancing (**b**): in red the seats available; measurements in millimeters

As it can be seen (Fig. 1), the introduction of interpersonal distancing on-board public transport is seen as one of the most effective measures. In fact, closed and crowded environments may increase the risk for transmission of COVID-19 [16] which occurs through droplets (5 to 10 μm) and aerosols (smaller than 5 μm) exhaled from infected individuals when breathing, speaking, coughing or sneezing [15]. However, there is still uncertainty about what is the optimal interpersonal distancing to prevent indoor contagion: studies argue that large droplets do not travel more than 2 m [1, 13]; other studies suggest that this distance may not be enough to exclude the possibility of contagion [17]. As a matter of fact, public authorities have been proposing interpersonal distancing ranging from 1 to 2 m, following either the advice of the World Health Organization suggesting one meter [21] or other health associations suggesting two meters [6]. In both cases, the impact on vehicle capacity is relevant: depending on vehicle layout and type of service (urban, suburban, ex-urban), the capacity reduction may drop to 20%–50% of full vehicle capacity (Fig. 2).

The vehicle capacity drop is the main reason for interpersonal distancing to be considered as highly effective but also highly unsustainable. In fact, it is difficult to be implemented on metro or bus services since it requires an access control system either on-board the vehicle or at the station, with additional costs for the PT operator. Moreover, when the demand flows exceed vehicle capacity, travelers might experience additional waiting time due to queues to access platforms and/or vehicles. The occurrence of these problems can either discourage travels or divert users to other modes of transport. This occurs also when the PT service is operated with mandatory seat reservation (e.g. on high-speed trains): here interpersonal distancing can be easily implemented by making available only suitably spaced seats (e.g. the red ones in Fig. 2), but this could lead to revenue losses when demand exceeds the number of available seats.

In principle, a way to counterbalance the reduction in vehicle capacity might be to increase service frequency. However, in many cases lines are already operated at full capacity (e.g. during peak periods): there is no spare infrastructure capacity or a lack of additional vehicles and drivers [20]. In any case, an increase of service frequency would have an additional cost for PT companies that could be unbearable. In fact, it has to be considered that operators have already experienced a significant drop in demand due to COVID-19, and consequently in ticketing revenues, together with an increase in sanitization and cleaning costs, in order to maintain the highest hygiene standards during service hours.

As a matter of fact, keeping the interpersonal distancing on-board vehicles can give rise to several critical issues that need to be assessed ex-ante in order to implement countermeasures to prevent discomfort to travelers and to possibly balance the financial losses for PT operators.

## Methodological approach

**Fig. 3:**
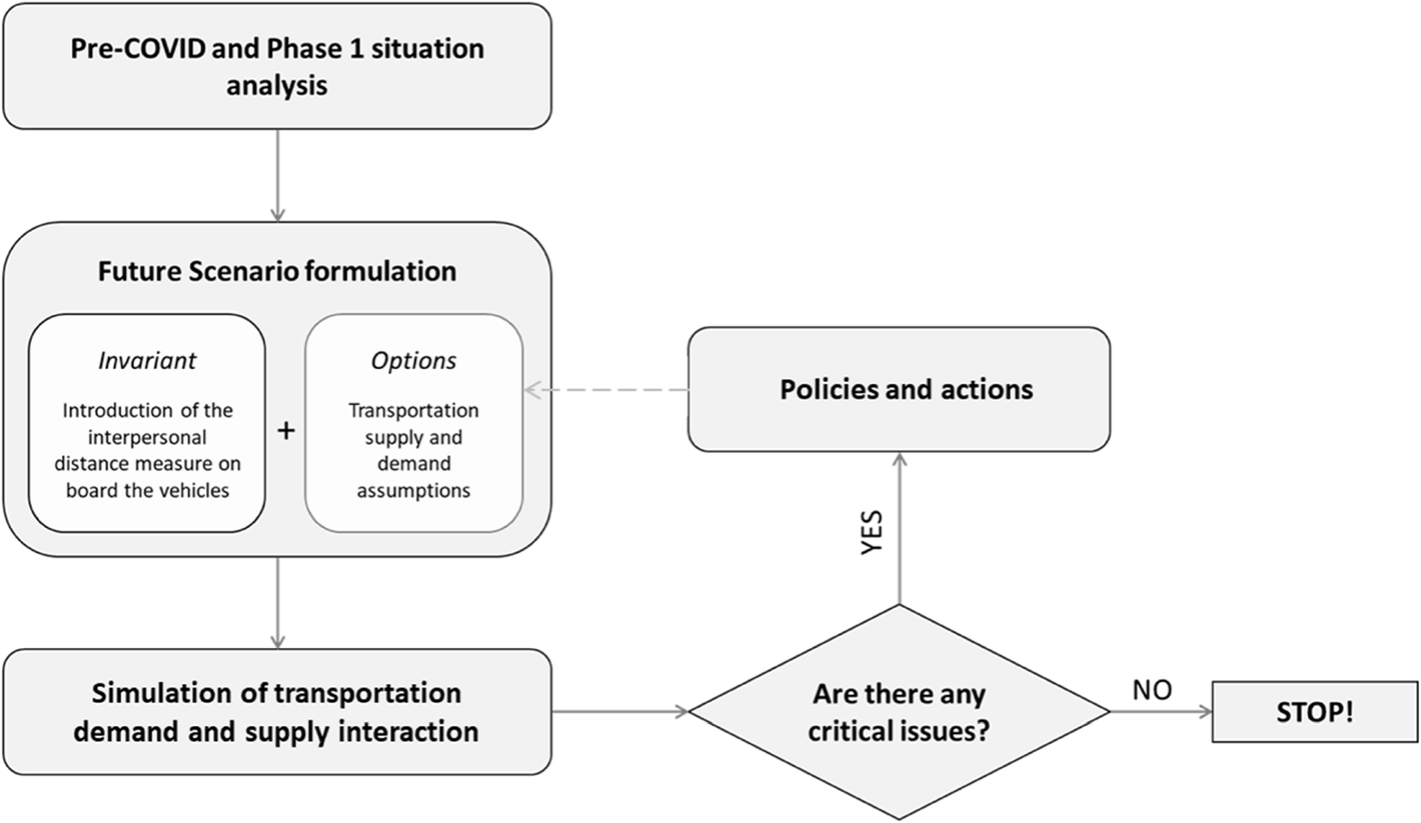
Schematic representation of the proposed methodology to identify PT system critical issues

The simulation-based approach schematically depicted in Fig. 3 is proposed to answer the above research question, i.e. under which condition of demand and supply the interpersonal distancing gives rise to any critical issues, either for the travelers or for PT companies.

The first step consisted in the analysis of two reference scenarios assumed as upper and lower benchmarks for the mobility conditions: the “Pre-COVID” scenario refers to November 2019 when there were no restrictions both on demand and supply, while “Phase 1” scenario corresponds to the first phase of the emergency (i.e. March–April 2020 in Italy) when only essential trips were allowed. With reference to these two scenarios, the following data were collected and analyzed:

- On the supply side: service frequencies and timetables, vehicle type, and seats’ layout on-board the vehicle;
- On the demand side: boarding and alighting passengers at stations on each single run.

The second step refers to the formulation of the future analysis scenarios where, on the one hand, some containment measures, such as the interpersonal distancing on-board vehicles, are assumed as invariant, and, on the other, several assumptions on transport supply (i.e. service frequency) and demand levels are tested.

The identified scenarios have been simulated by means of a schedule-based assignment model [24], allowing to estimate on-board flows on each single train during the day.

The schedule-based approach requires both a diachronic graph representation of the timetable of the daily runs and a segmentation of travelers according to the time they wish either to start their journey or to arrive at their destination. In the literature, these are respectively termed as Desired Departure Time (DDT) and Desired Arrival Time (DAT) [4]. Generally, travelers are segmented by DDT at the origin (e.g. home) or by DAT at destination (e.g. work) depending on trip purpose. For sake of simplicity, DATs are shifted back in time by a quantity equal to the travel time on the chosen run in order to be transformed into DDTs. The distribution of demand by Desired Departure Time, *d*_*DDT*_, has been estimated based on the available boarded/alighted passenger counts at each station.

For a given DDT, travelers’ choice set *C* consists of all the runs departing within a 60 min long temporal centered in the DDT: for instance, if the DDT of a user is 8:30 am, the choice set would consist of all the runs departing between 8:00 and 9:00 am. A random utility model has been adopted to simulate the choice among the runs *j* in the choice set *C* [2]. The perceived utility *U*_*j*_ of each alternative *j* is given by the sum of a systematic utility *V*_*j*_ and of a random residual *ε*_*j*_, assumed as independently and identically distributed as a Gumbel random variable with zero mean and scale parameter θ (i.e. Logit model). Under these assumptions the probability of choosing the generic run *j* among all the alternatives in the choice set *C* is the following one:

$$ p\left[j| DDT\right]=\frac{\exp \left({V}_{j\mid DDT}/\theta \right)}{\sum_{\mathrm{i}\in \mathrm{C}}\exp \left({V}_{i\mid DDT}/\theta \right)} $$

The systematic utility *V*_*j*_, defined in linear form as a weighted sum of attribute *X*_*kj*_ by means of *β*_*j*_ parameters, is given by:

$$ {V}_{j\mid DDT}\left({\boldsymbol{X}}_j\right)={\beta}_T\bullet {T}_j+{\beta}_{LDP}\bullet {LDP}_{j\mid DDT}+{\beta}_{EDP}\bullet {EDP}_{j\mid DDT} $$

where:

- *T*_*j*_ is the travel time of the run *j*;
- *LDP*_*j*_ is the late departure penalty relating to run *j*;
- *EDP*_*j*_ is the early departure penalty relating to run *j*.

For a given alternative *j*, the early/late departure penalties are calculated by the difference between the Desired Departure Time (DDT) and the actual run departure time. For a given run with departure time *ω*_*j*_ it results:

- ▪ if *ω*_*j*_ < *DDT* (i.e. run *j* departure time is earlier than the Desired Departure Time, DDT): *EAP*_*j*_ = *DDT* − *ω*_*j*_ and *LDP*_*j*_ = 0
- ▪ if *ω*_*j*_ > *DDT* (i.e. run *j* departure time is later than the Desired Departure time, DDT): *EAP*_*j*_ = 0 and *LDP*_*j*_ = *ω*_*j*_ − *DDT*
- ▪ if *ω*_*j*_ = *DDT* (i.e. run *j* is scheduled exactly at the Desired Departure Time, DDT): *EAP*_*j*_ = 0 and *LDP*_*j*_ = 0

The flow on each run, *f*_*j*_, is finally given by summing up over DDT the demand flows times the probability of choosing the specific run *j*:

$$ {f}_j={\sum}_{DDT}{d}_{DDT}\bullet p\left[j| DDT\right] $$

The flows on each run are assigned to the diachronic graph to get an estimate of the flow on each section of the run. In the case of low frequency and uncongested public transport networks, a DNL (Dynamic Network Loading) model can be used: an exhaustive review of such models is reported in Wilson and Nuzzolo [24]. In our case studies, a stochastic network loading model with no capacity constrains has been adopted to estimate the link flows on the diachronic network.

The estimated flows on the sections of the individual runs/train are compared with trains capacities to identify critical conditions of overcrowded vehicles or unsatisfied demand. In such cases, policies or actions to solve them are identified and simulated again.

## Results

Two case studies with a different service access system have been considered: a suburban railway line where no seat reservation is required, and a High-speed Rail (HSR) line for which seat reservation is mandatory. Different criticalities might be arising in the two cases: in the former, (suburban line case) trains could get overcrowded and therefore be not compliant with the interpersonal distancing rules; in the second case (HSR line) part of the potential demand might be unsatisfied due to seat unavailability, and, in turn, be shifted to other modes with consequent revenue losses for the operators.

### The case of the suburban railway line S13

**Fig. 4:**
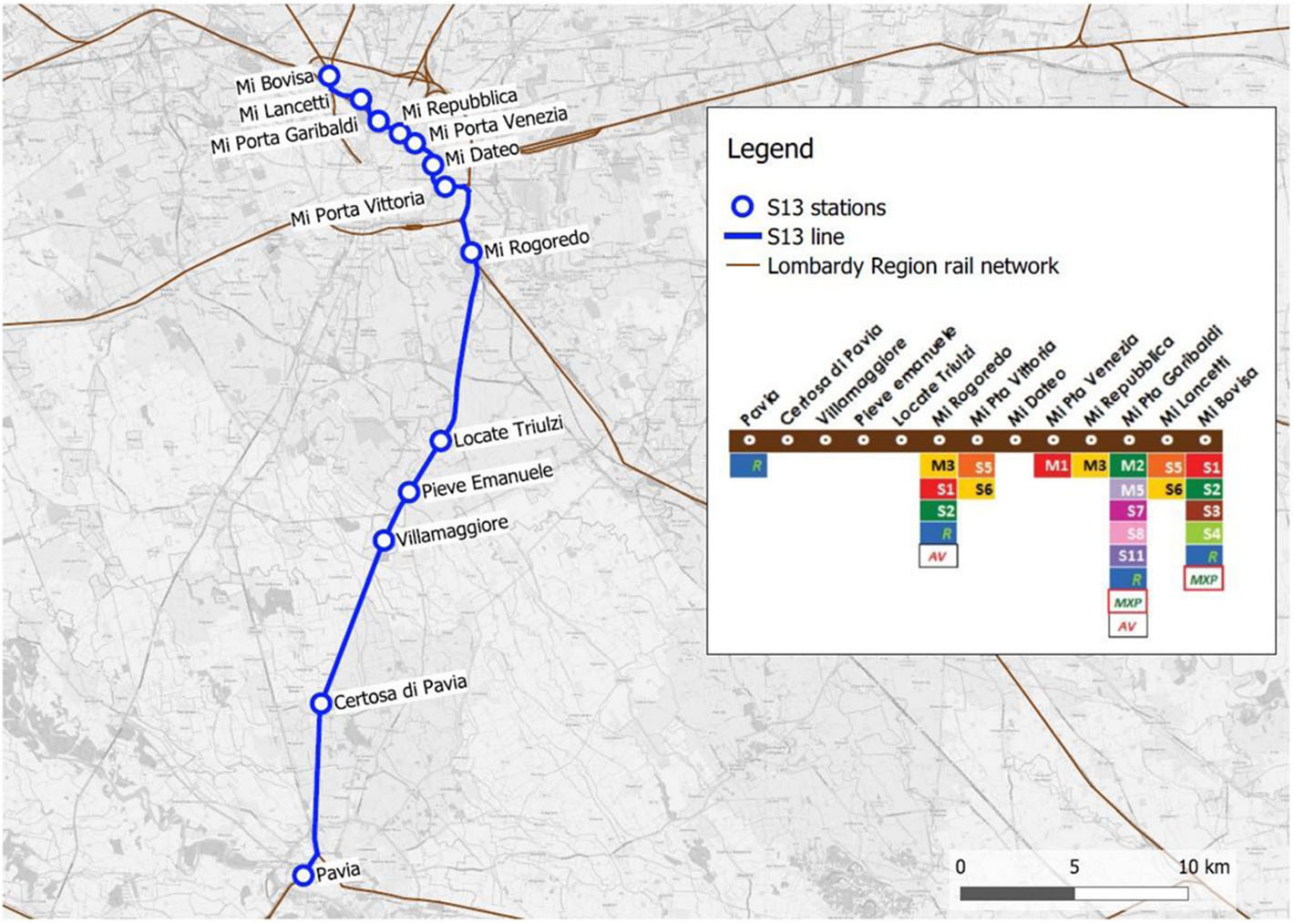
The S13 suburban railway line and its interconnections with metro (M) and other suburban (S) lines

The S13 suburban railway line connects Bovisa (Milan) with Pavia, crossing the so called “Passante Ferroviario”, a railway track that joins lines coming from the north-west with those coming from the east and south-east, by going mainly under the city center of Milan. The S13 line provides a direct link between Pavia and some areas (the city center and the northern territory) that fall within the municipality of Milan, passing through the Rogoredo hub. From Rogoredo station, in fact, trains enter the “Passante Ferroviario”, intercepting all four metro lines and making a total of seven urban stops before reaching Bovisa station. Figure 4 shows in detail the line route, including stops and interconnections with other PT services.

**Fig. 5:**
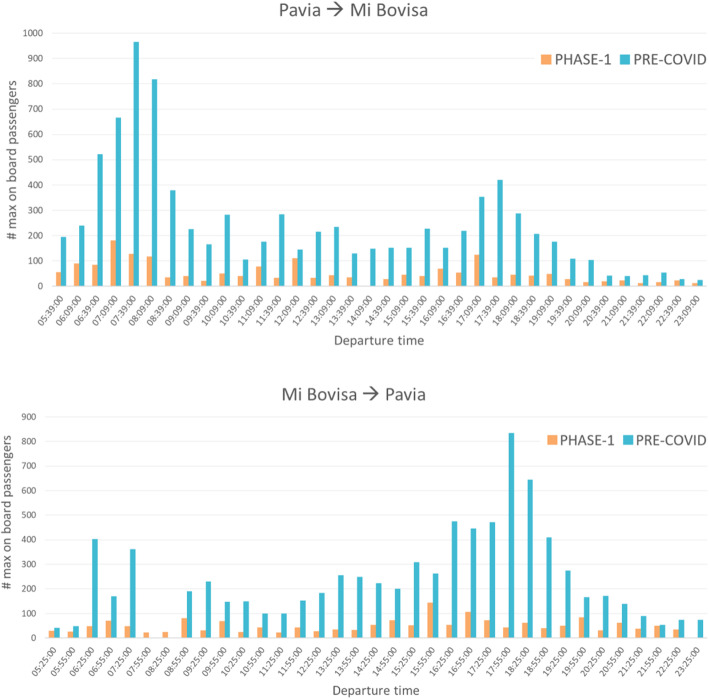
Maximum number of on-board passengers for each S13 run during a working day (Pavia – Mi Bovisa and Mi Bovisa – Pavia directions)

From the analysis of the Pre-COVID and Phase 1 scenarios, a reduction of the maximum number of on-board passengers in a working day of about 79% was observed for both directions, Pavia-Mi Bovisa and Mi Bovisa – Pavia (Fig. 5). However, the structure of the demand did still present the same two peaks periods: a morning peak (from 6:30 to 9:00 am, on the Pavia – Mi Bovisa direction) and an evening peak (from 17:00 to 19:00 on the Mi Bovisa-Pavia direction).

In addition to simulating the Pre-COVID and Phase 1 scenarios, the following scenarios have been considered:

- **PARTIAL RE-OPENING**, that considers a partial reopening of universities and schools, i.e. half of the students would be attending in presence and half in distance mode, resulting in a demand level for study purposes of 50% less than the one in the Pre-COVID scenario;
- **NO-STUDENTS**, that assumed schools and universities being closed and teaching activities performed entirely in distance mode, thus resulting in a null demand for study purposes in order to reduce trains loads, in particular during the peak periods.

On the supply side, both the service frequency, i.e. the number of trains per day and during peak periods, and the type of rolling stock were considered as invariant. With reference to the latter, a TSR (Treno Servizio Regionale) train, composed of five coaches, two with a cab and a wheelchair bay for disabled people (MCH) and the others without (M), was simulated. A full train capacity, including all the available seats and considering an occupancy rate of 4 passengers per square meter for the standing places, comprises 960 passengers/train. The introduction of interpersonal distancing measures yields a train capacity of 270 passengers, assuming half of the seats available plus an aliquot of standees equal to 10% of the seats available in normal conditions.

**Fig. 6:**
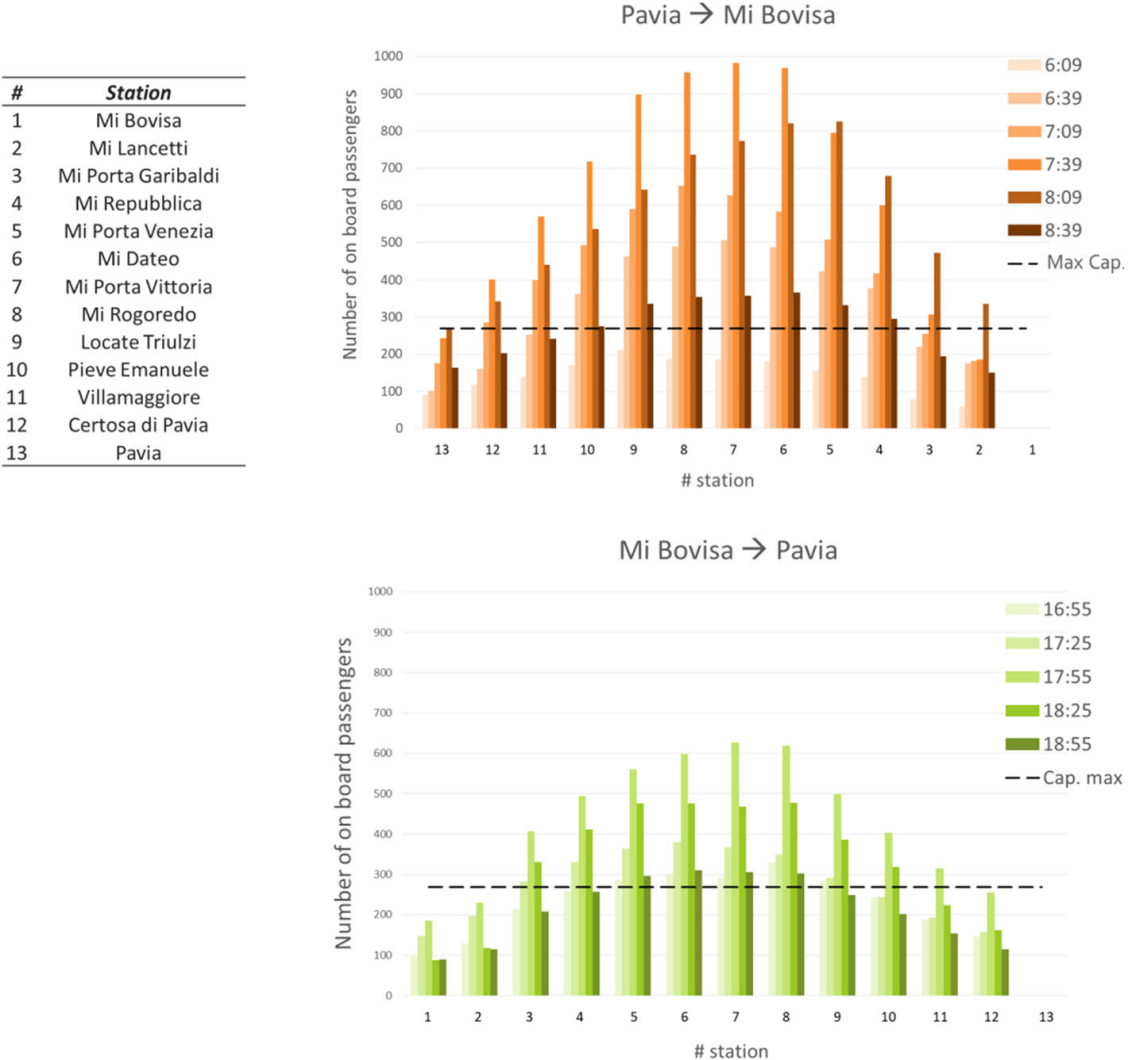
Flow diagram of passengers on-board S13 line train during the morning peak in the Pavia–Bovisa direction and in the evening peak in the Bovisa-Pavia direction (Pre-COVID scenario)

For each simulated scenario, the estimated station-to-station OD matrix referred to the peak periods has been assigned in order to estimate the flows on the trains operating in the considered morning (6:30–9:00) and evening (17:00–19:00) peak periods. Results are shown in Fig. 6.

With the introduction of interpersonal distancing, a severe overload of the trains in almost all the stations and for almost all the trains running in the peak periods can be observed.

Table 1 reports the aggregate results for the simulated scenarios, in terms of number of stations with departing trains in overcrowded conditions and of average number of passengers exceeding train capacity: even assuming zero trips for study purposes, there would be a number of stations (6 and 5 out of 13, respectively on the Pavia – Mi Bovisa and Mi Bovisa - Pavia directions) with departing trains exceeding the allowed capacity of 270 passengers, with an average number of overcrowding ranging between 12 and 47 passengers/train.

**Table 1:** Outputs of the scenario simulations with capacity restrictions (i.e. 270 passenger/train) under different assumptions of demand levels

	Demand level			
	PRE-COVID	PARTIAL RE-OPENING	NO-STUDENTS	PHASE 1
**Pavia – Bovisa (6:30–9:00)**				
Number of stations with oversaturated trains (# stations)	11	9	6	0
Average number of passengers exceeding train capacity (pax/train)	157	116	47	0
**Bovisa – Pavia (17:00–19:00)**				
Number of stations with oversaturated trains (# stations)	9	7	5	0
Average number of passengers exceeding train capacity (pax/train)	102	48	12	0

### The case of the HSR lines: Milan-Rome-Naples

The Milan-Naples high-speed Rail (HSR) line connects some of the most attractive Italian cities, such as Bologna, Florence and Rome [5]. In this study the direct services between Rome-Milan and Rome-Naples have been considered.

**Fig. 7:**
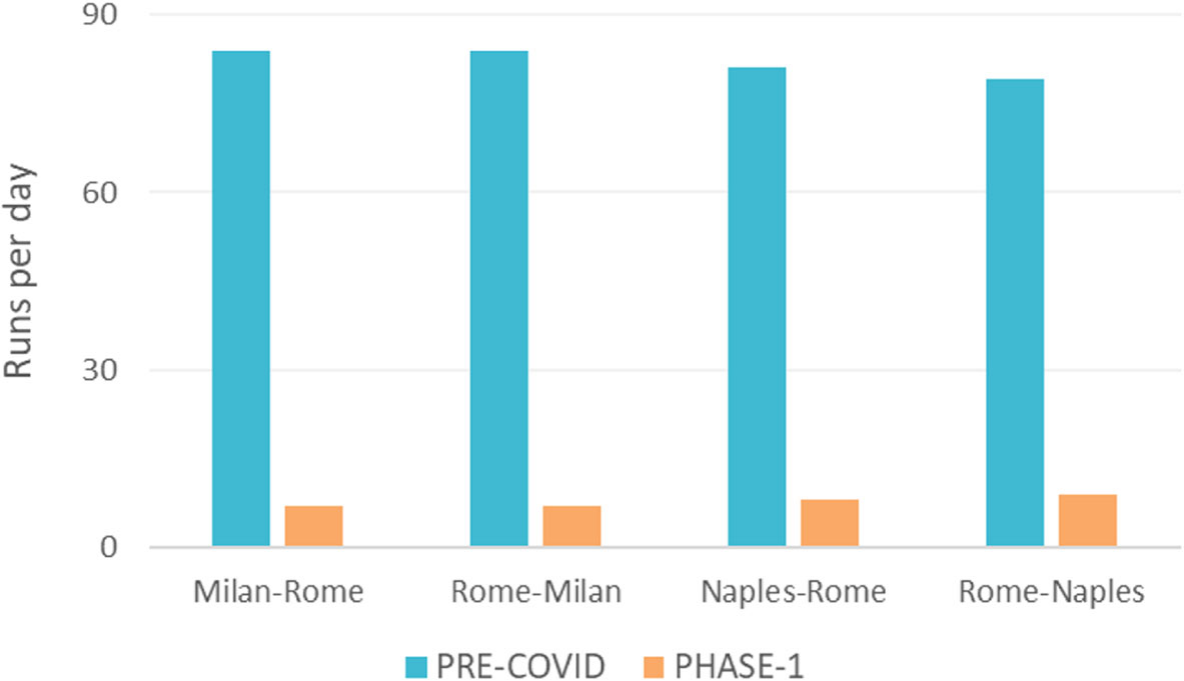
Comparison of runs per day between the PRE-COVID and the PHASE-1 scenario for the different origin-destination pairs under analysis

The analysis of the Pre-COVID and Phase 1 scenarios was based on the timetable of HSR services offered by the two HSR undertakings, i.e. the incumbent Trenitalia and NTV (Nuovo Trasporto Viaggiatori): a decrease of about 90% of services per day was observed from the Pre-COVID to the PHASE 1 scenario (Fig. 7).

With reference to the rolling stock, the two undertakings use different types of train: ETR500 and ETR1000 trains for Trenitalia, with a capacity of 597 and 467 seats, respectively; AGV575 and ETR675 trains for NTV, with capacities of 462 and 472 seats, respectively. In the simulated scenarios, a capacity reduction of 50% of the seats available was considered due to the introduction of interpersonal distancing measures. Moreover, HSR service frequency has been assumed equal to 24 trains/day on the Milan-Rome line, and equal to 29 trains/day on the Rome-Naples one.

On the demand side, in addition to the Pre-COVID scenario, two scenarios have been simulated which respectively consider 50% and 40% of the Pre-COVID demand level.

**Fig. 8:**
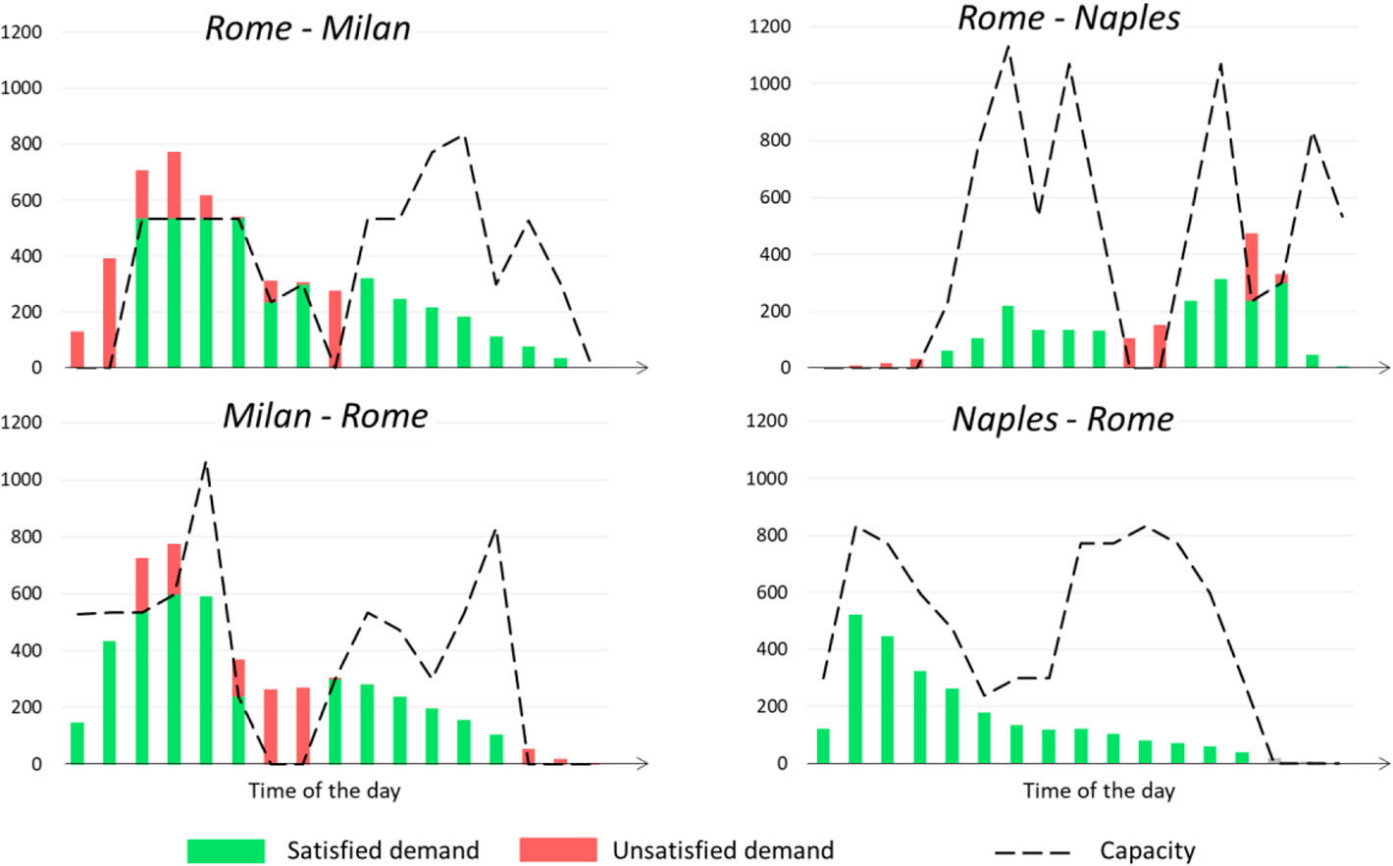
Estimated demand flows by hour-of -day in “SCENARIO 40%”

Figure 8 shows some of the outputs of the supply-demand interaction: for instance, the estimated demand flows in the Scenario 40%, disaggregated by hour-of-day, including those users that could not travel on HSR due to seats unavailability (i.e. unsatisfied demand). It can be observed that on the Rome-Milan and Milan-Rome directions there is quite a significative loss of passengers, respectively 24% and 20% (i.e. unsatisfied demand), whereas on the Rome-Naples line this is limited only to the Naples to Rome direction, with a loss of 17%.

Table 2 shows the unsatisfied demand by section and direction in the simulated scenarios. It can be observed that:

- In all the simulated scenarios, the most affected sections are those connecting Rome and Milan;
- The unsatisfied demand is very significant when the volumes are assumed equal to those prior to the pandemic (Pre-COVID), with a peak of 62% on the Rome-Milan direction;
- In the reduced demand scenarios, the unsatisfied demand is between 20% and 30% on the Rome-Milan-Rome line, and between 0% and 20% on the Rome-Naples-Rome line.

**Table 2:** Percentages of passengers lost by HSR undertakings, in the simulated scenarios, for each direction

		Rome - Milan	Milan - Rome	Rome - Naples	Naples – Rome
**Demand levels**	PRE-COVID SCENARIO (100%)	62%	49%	38%	16%
	SCENARIO 50%	30%	25%	19%	0%
	SCENARIO 40%	24%	20%	17%	0%

## Discussion

The simulations presented in the previous section were carried out for two different case studies: a suburban railway line and a HSR line, differentiated by demand characteristic (urban vs. ex-urban) and by the seat access system (e.g. free access vs. reservation required).

In the urban case, the results of the simulation show that keeping one-meter interpersonal distancing on-board vehicles can be sustainable only with drastic limitations to travels and activities (for example, those implemented during the Phase 1 of the pandemic emergency).

To cope with demand flows exceeding the allowed number of passengers on board the trains, an increase of line capacity up to 70%–80% is needed, meaning that no interpersonal distancing would be maintained for certain section of the lines, particularly, in the peak periods. This could be possible either by increasing the train capacity with an additional number of coaches per train, or by increasing line frequency in the peak periods. However, on the one hand, increasing the number of coaches per train could result in a train length greater than the length of the station platforms, that would be unfeasible. On the other hand, provided that the railway infrastructure capacity is typically close to the saturation during peak hours, the margin for increasing frequency is very limited. As a matter of fact, increasing line capacity, while keeping the interpersonal distancing on-board, could be achieved only with significant investment in new rolling stock and in the rail infrastructure, both requiring a long time for being achieved.

With reference to crowding issues at stations or at stops, in order to minimize the physical interactions between passengers, thus reducing public health risks, PT companies could implement a number of measures to keep users’ flows separate, using, for instance one-way entries or dedicated pathways. PT operators could also intervene on the procedures for getting on or getting off the train, thereby reducing crowding caused by interference between travellers walking in opposite directions, with the introduction of dedicated doors for boarding and alighting passengers. However, it has been shown that this measure could increase dwell times at stops, in particular if usually all doors were previously being used for getting on and off vehicles [12].

Alternative actions that could be implemented to mitigate crowding are:

- The introduction of advanced traveler’s information systems to monitor in real time trains/stations inflows and outflows, and to inform travelers about possible service unavailability or presence of queues due to overcrowding, using both electronic displays installed at bus stops / stations and mobile travel apps (see, for instance, [9]);
- The introduction of seat reservation systems to access trains and/or stations, similar to those for ex-urban services.

Making seat reservations via smartphone apps would also have the merit of reducing contacts and interactions among people with respect to purchasing tickets at offices. However, if on the one hand, these measures could help to manage the demand in peak periods, on the other hand, in case of recurrent over-crowded trains, they could induce a modal shift towards other modes and services, due to the unreliability of service travel times (in particular, waiting times). In addition, they could penalize those travellers not familiar with the use of digital devices (e.g. the elderly), leading them to be more incline to select modes of transport that do not require such procedures/restrictions.

From a system perspective, more radical solutions to be implemented by public authorities could be those of:

- Reducing demand levels by promoting working from home and/or distance learning for school and university students; this measure would undoubtedly reduce the mobility volumes, but also ticketing revenues for PT operators, thus requiring additional public subsidies to maintain the same transport supply levels or fares;
- Smoothing the demand peaks by spreading the demand volumes over the day; this measure could be implemented by changing school timetables, by introducing differentiated schedules for office and factory working hours, by changing opening times in the cities (e.g. for shopping, public services, medical care, ...). This would induce deep changes in household habits and daily routines, probably generating in some cases strong opposition by communities or workers’ associations, thus requiring strong political commitment and administrative actions.

In the ex-urban case, the simulation has shown that keeping interpersonal distancing on board HSR train means halving capacity and, with reference to our case study, even with demand levels of 50% or 40% of those during PRE-Covid times, there would be quite a significant number of unsatisfied demand due to seat unavailability, especially in peak periods. This would imply a significant loss for the railway undertakings in terms of ticketing revenues (i.e. in a range of 20%–60% of those before the health emergency). Furthermore, this could induce a significant modal shift towards less sustainable modes of transport (car and air) in the short term. The behavioural changes in the choice of transportation means could become structural due to inertial individual factors and very difficult to be reversed in the long period, unless strong incentives such as discounted fares are introduced.

To partially contrast this situation, railway undertakings can redesign vehicle layout, by installing barriers and dividing walls between seats in order to increase train capacity while keeping safe interpersonal distancing. However, this measure in any case, cannot prevent entirely their ticketing losses that could have direct impact on their budget and labor productivity, but also indirect ones on the HSR system performances. In Italy, for example, the competition between the incumbent Trenitalia and the private undertakings NTV (Nuovo Trasporto Viaggiatori) that generated significant benefits for travelers in terms of fares and services [3] could be undermined if the private undertaking abandoned the HSR market owing to the risk of no profit or deficit budget.

Given the strategic importance of the rail sector and of keeping the competition in HSR market, public authorities could introduce some compensation (e.g. in the form of reductions of the fees for the use of the infrastructure) to support HSR undertakings and to sustain competition in the HSR market. This could avoid impacting service quality, the increase in fares for travelers and modal shifts, in particular towards private vehicles, which would lead to a high level of road congestion, with severe consequences on the environment, both in terms of landscape and air quality.

## Conclusion

This paper focuses on the introduction of interpersonal distancing on-board vehicles, proposed as a community containment measure to cope with the COVID-19 pandemic. A simulation-based procedure is proposed to assess the impact of such measure, identifying criticalities and possible complementary actions to mitigate its direct and indirect impact.

The applications to the two case studies in Italy of a suburban railway line and an HSR line, differentiated by demand characteristics (urban vs. ex-urban) and seat access system (e.g. free access vs. reservations required) have shown the unsustainability of the measure in the medium-long period. In fact, in the presented urban case, to cope with demand flows exceeding the allowed number of passengers on board the trains, particularly in the peak periods, an increase of line capacity up to 70%–80% is needed, that could be achieved using (wherever possible) longer and more frequent trains. On the other hands, in the (HSR) ex-urban case, a loss for the railway undertakings in terms of ticketing revenues has been estimated in a range of 20%–60% of those before the health emergency, depending on the demand daily temporal profile.

New policies should be not only limited to the transport domain, but also promote working from home and/or distance learning as well as change and differentiate opening times in the cities (e.g. for schools, industries, shops, etc.) in order to manage the demand peaks at the stations and on-board vehicles. Moreover, the results have shown the need of public subsidies for the railways undertakings and PT companies to cope with the ticketing revenue losses suffered, and to maintain service quality levels.

The simulation results have confirmed the perception expressed by a panel of PT operators according to which keeping interpersonal distancing is unsustainable in the medium-long term without any compensating measures [7].

Further insights and developments of this study may deal with the application of the proposed methodology to an extended transport network, with an overall analysis of the criticalities and their spatial distribution. In particular, the method can be useful to assess the potential modal shift towards private vehicles and to quantify the financial impact on the PT companies’ budget.

## Data Availability

Most datasets used and/or analyzed during the current study are available from the corresponding author upon reasonable request. Restrictions apply to the availability of data that was used under license for the current study, and therefore are not publicly available. This data is however available from the authors with permission of the Trenitalia e Trenord companies.
